# Successful thrombolysis following enoxaparin therapy in two pediatric patients with congenital heart disease presenting with intracardiac and cerebral thrombosis

**DOI:** 10.1186/1477-9560-12-19

**Published:** 2014-09-09

**Authors:** Gesa Wiegand, Vanya Icheva, Martin Schöning, Michael Hofbeck

**Affiliations:** 1 Department of Pediatric Cardiology, University Children’s Hospital, Hoppe-Seyler-Strasse 1, 72076 Tuebingen, Germany; 2 Department of Pediatric Neurology, University Children’s Hospital, Tuebingen, Germany

**Keywords:** Enoxaparin, Low-molecular-weight heparin, Children, Thrombosis

## Abstract

Enoxaparin displays fibrinolytic activity through stimulation of endothelial release of tissue plasminogen activator. Moreover, enoxaparin increases the release of tissue factor pathway inhibitor, which inhibits coagulation activity. However, there are only few reports regarding the use of enoxaparin for the treatment of children with thrombosis complicating congenital heart disease. We report the clinical findings from two patients, one child with an A. cerebri media infarction and another with a left ventricular thrombus. In both cases successful thrombolysis was obtained by intravenous administration of enoxaparin. The first patient was a 12-year-old girl with an atrioventricular septal defect, who underwent biventricular repair at the age of 8 months. She presented with right-sided middle cerebral artery infarction. Thrombolysis was contraindicated, because she was beyond the therapeutic window recommended by accepted guidelines. Enoxaparin 2.5 mg/kg/d was administered as a continuous intravenous infusion (CII). The MRI 10 days later revealed a reopened middle cerebral artery and she experienced complete remission of the neurological signs. The second patient was a 16-year-old boy who had tetralogy of Fallot corrected in late infancy. He presented with severe heart failure and a mural thrombus in the left ventricular apex. Enoxaparin was administered and resulted in complete disappearance of the thrombus within a week. According to our experience, CII of enoxaparin was safe and well tolerated without secondary bleeding and resulted in complete dissolution of the thrombi without secondary embolization. Therefore, CII of enoxaparin may be a possible alternative for the treatment of thrombotic complications in children with contraindications against conventional thrombolytic therapy.

## Background

In the pediatric population, the treatment of venous thromboembolism (VTE) is hampered by a lack of adequate pediatric guidelines or large-scale pediatric studies. Until recent years, the administration of unfractioned heparin (UFH) followed by an oral anticoagulant was regarded as the therapy of choice for the treatment of VTE in children. However, low-molecular-weight heparin (LMWH), especially enoxaparin, since the early nineties is increasingly used in the pediatric population [1-5] and is recommended by the guidelines of the American College of Chest Physicians [6]. Nevertheless it is necessary to mention, that neither the subcutaneous nor the intravenous application of enoxaparin is approved for use in children. Therefore written informed consent of the parents is recommended prior to application.

Enoxaparin has a fibrinolytic activity through stimulation of endothelial release of tPA (tissue plasminogen activator) [7]. Moreover, enoxaparin increases tissue factor pathway inhibitor (TFPI) release, which inhibits coagulation activity [8]. However, only few reports describe the use of enoxaparin via continuous intravenous infusion (CII) [9-11]. As substantial variability of the plasmatic anti-Xa concentrations were being observed during standard subcutaneous administration of the recommended dosages, *Feng et al*. introduced the CII route to increase the efficiency of the therapy. Therefore, they evaluated the pharmacokinetic profile and defined a dosage strategy for administering enoxaparin by CII even in patients with altered renal function [9]. *Kane-Gill et al.* also reported about administration of enoxaprin as CII to evaluate the influence of renal function on enoxaparin activity [10]. *Lorenzini et al.* mentioned two patients who received the drug by CII while being on renal replacement therapy (hemofiltration) for 15 and 60 days [11].

So far, however we identified no mention in the literature of this therapy for the treatment of children with thrombosis complicating congenital heart disease.

### Case presentation

**Figure 1:**
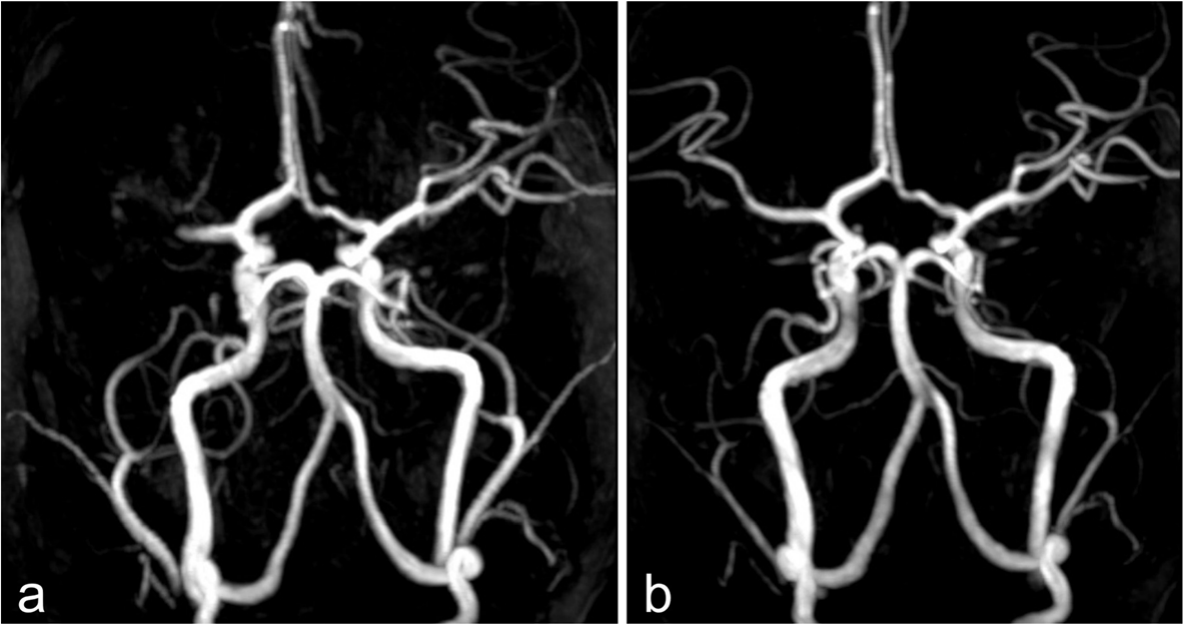
**Cerebral arteriogram showing a right-sided middle cerebral artery infarction (a).** Following thrombolytic therapy with enoxaparin, the MRI revealed normal perfusion of the cerebral arteries **(b)**.

The first patient (patient 1) was a 12-year-old girl with a complex congenital cardiac malformation (heterotaxy syndrome including atrioventricular septal defect and partial anomalous pulmonary venous return), who underwent biventricular repair at the age of 8 months. At the age of 12 years, the girl presented with left-sided hemiparesis. Magnetic resonance imaging (MRI) revealed a right-sided middle cerebral artery infarction (Figure 1a) with infarct demarcation in the right hemispheric white matter. Conventional thrombolysis with rtPA was contraindicated because she presented more than 6 hours after symptoms commenced, which was beyond the therapeutic window recommended for the adult population according to accepted guidelines [12].

Transoesophageal echocardiography was unremarkable with no intracardiac thrombus formations.

**Figure 2:**
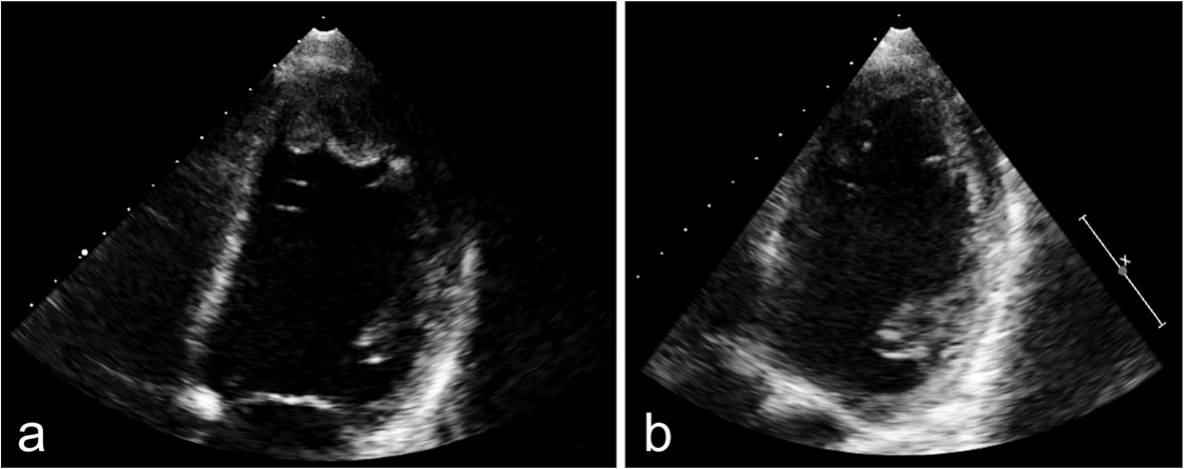
**Echocardiography of the left ventricle shows thrombus formation in the apex (a).** Following thrombolytic therapy with enoxaparin, echocardiography revealed complete disappearance of the thrombus **(b)**.

Enoxaparin (2.5 mg/kg/d) was administered by CII (anti-Xa levels 0.6–1.0 IU/ml). Hereby we calculated the dosage of enoxaparin according to the guidelines of the American College of Chest Physicians [6] with age specific dosages of 1–1.5 mg twice daily, respectively 2–3 mg/kg/d. We used enoxaparin sodium as pre-filled syringes, which was diluted in 50 ml saline 0.9% and was changed every 24 hours, since the physical and chemical stability was verified for periods longer than 24 h at a temperature of 21°C [13]. Within a few days the girl experienced complete remission of the clinical symptoms and 10 days later the MRI revealed a patent middle cerebral artery (Figure 1b). After 4 days, enoxaparin was switched from CII to a subcutaneous administration with anti-Xa levels still kept above 0.6 - 1 IU/ml. The switch of the administration route was decided in view of the disappearance of the clinically relevant hemiparesis and a normal transcranial doppler profile. D-dimer levels (normal range: 0–0.23 μg/ml) were not elevated during therapy with a maximum of 0.06 μg/ml measured on the first day.The second patient was a 16-year-old boy (patient 2) who had tetralogy of Fallot corrected in late infancy. The patient reported progressive fatigue for one month and recurrent episodes of stomach ache. Echocardiography revealed severe heart failure with an enlarged and poorly contracting left ventricle, a moderate mitral valve insufficiency and a mural thrombus formation in the left ventricular apex (Figure 2a). Enoxaparin was administered at 2 mg/kg/d as a CII, in addition to inotropic medication with dobutamine and levosimendan, amongst others. Due to a modest elevation of anti-Xa levels, enoxaparin was increased to 4 mg/kg/d for a few hours. The thrombus diminished in size and complete dissolution occurred within one week (Figure 2b). During this therapy there was no evidence for thrombus embolization and a cranial CT scan discovered no evidence of intracranial hemorrhage. A chest CT scan confirmed the diagnosis of cardiomegaly and hypodense structures in the apex of the left ventricle correlated with thrombus formation. After 7 days the CII was switched to a subcutaneous administration with similar anti-Xa levels and after 2 weeks the antithrombotic therapy was switched to acetylsalicylic acid because of expected poor compliance in a difficult family situation. During enoxaparin treatment, d-dimer levels increased to a maximum of 5.1 μg/ml after 2 days of therapy.

Cardiac catheterization including myocardial biopsy was performed and showed that the etiology of the severe heart failure was dilative cardiomyopathy of unknown origin. The CII of enoxaparin was paused 4 hours prior to the invasive procedure.

To obtain a more efficient thrombolysis, anti-Xa levels were raised to the upper limits of the recommended therapeutic levels (between 0.7 and 1.0 IU/ml [7]).

Platelet counts were monitored daily. None of the patients had renal dysfunction or manifested signs of heparin induced thrombycytopenia (HIT).

Routine thrombophilia screening, including APC resistance, factor II mutation, protein C and S, antithrombin, homocysteine, lipoprotein (a), antiphospholipid antibodies and factor VIII, was negative in both patients prior to the enoxaparin therapy.

## Discussion

**Figure 3:**
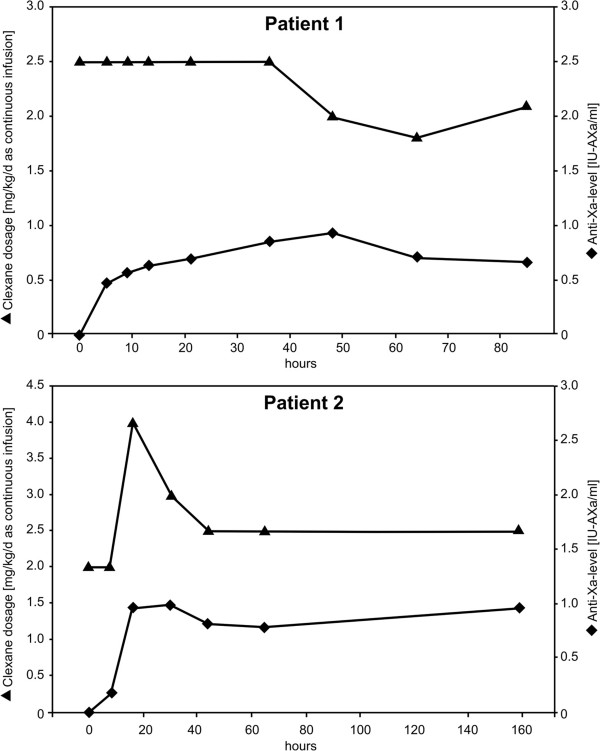
Anti-Xa levels of patient 1 (a) and patient 2 (b) in correlation with the administered enoxaparin (Clexane) dosage.

LMWHs are prescribed for the treatment and prevention of several conditions including deep venous thrombosis, pulmonary embolism, acute coronary syndromes and atrial fibrillation [10,14,15]. The standardized method of administration for LMWH, including enoxaparin, is by subcutaneous injection, but *Kane-Gill et al.* found that the safety of enoxaparin administered by CII was comparable to that of subcutaneous administration [10]. Intravenous application has also been described in patients during percutaneous coronary interventions [16]. Few reports exist on the use of LMWHs by CII in the setting of deep vein thrombosis, hemodialysis and pulmonary embolism [11,17], and there is even less information regarding the use of LMWH administered as a CII in children [18]. In both of our patients, we started with an initial dosage of 2–2.5 mg/kg as a CII. Despite being aware of the fact that the peak drug level is reached 4 hours following subcutaneous administration, we undertook a first sampling of the anti-Xa levels 6–8 hours after the start of the infusion. We chose to wait that long in order to obtain a steady-state, especially in view of the fact that despite administering the same dosage over several days in the setting of CII the anti-Xa levels seemed to increase further. Therefore, daily blood sampling was performed because anti-Xa levels - as we expected - continued to rise, especially during the first two to three days. In both patients, a therapeutic level of 0.6–1.0 IU/ml was obtained with a dosage of 1.8–2.5 mg/kg/d (Figure 3a and b). Administration of enoxaparin for “thrombolysis” via CII can result in a more constant and predictable anticoagulation than subcutaneous injection [10].

During the treatment, d-dimer levels of the patient 2 increased to a maximum of 5.1 μg/ml after 2 days of therapy. In combination with a thrombus regression seen on echocardiography, we consider this as a sign of thrombus regression by thrombolysis.

Enoxaparin has a predominantly renal clearance [19]. Renal dysfunction was suggested to be involved in elevated anti-Xa levels, and subsequently in bleeding complications [9]. This association was not confirmed by others [10]. Anti-Xa clearance in ICU patients is about half of their non-ICU counterparts, caused by altered enoxaparin pharmacokinetics in the critically ill [10,20]. Therefore, in the initial phase of the therapy anti-Xa levels should be monitored daily, and, according to the manufacturer, dose reduction applied for creatinine clearance values of below 30 ml/min.

In the literature, reported undesirable side effects caused by CII of enoxaparin include gastrointestinal bleeding, epistaxis, bleeding from the oral mucosa, thrombocytopenia and wound bleeding, none of which correlate with high anti-Xa levels [10]. In our two patients, however, there were no adverse reactions.

When compared to other established LMWH drugs, enoxaparin shows the highest anti-Xa activity, the highest anti-thrombin effect and the longest biological halflife [8]. Antithrombotic effects are the result of inhibition of the tissue factor/factor VIIa complex and the release of TFPI. However, enoxaparin possesses profibrinolytic qualities such as the ability to significantly increase tPA release from endothelial cells [7,8].

According to our experience, intravenous administration of enoxaparin as a CII appears to be safe and well tolerated without bleeding complications. In both patients, CII of enoxaparin resulted in complete dissolution of the thrombi without secondary embolization. Therefore, CII of enoxaparin may be a possible alternative treatment for thrombotic complications in children with contraindications against conventional thrombolytic therapy.

Based on the data of our two patients, anti-Xa levels were higher when the same therapeutic dosage of enoxaparin (2 mg/kg/d) was administered by CII than by subcutaneous administration. Similar observations were reported by *Sanchez-Pena et al.* who found that anti-Xa activities were almost doubled when enoxaparin was administered intravenously rather than subcutaneously [16]. Additionally, in contrast to subcutaneous administration, where anti-Xa levels reflect only the maximal peak activity, CII of enoxaparin results in more stable and predictable anti-Xa levels, which lead to more consistent anticoagulant effects.

CII of enoxaparin is advantageous, particularly for children who cannot be treated at home, because it avoids repeated painful injections, which may improve the patient’s quality of living. Furthermore, CII also allows application via a peripheral venous catheter. Another advantage is evident in children with very low weight, because it is often difficult to give a small dose subcutaneously due to the very small amount of medication injected [18].

## Conclusion

CII of enoxaparin is as safe as subcutaneous administration and can be safely used for both thrombolysis and prophylaxis in hospitalized patients with a wide range of thrombotic conditions.

Pharmacokinetic studies in the paediatric population and further fine-tuning of the dosage regimens in children will be evaluated in future.

### Consent

Written informed consent was obtained from the patients for publication of both case reports and any accompanying images. A copy of written consent is available for review by Editor-in-Chief of this journal.

## Abbreviations

CII: Continuous intravenous infusion; VTE: Venous thromboembolism; UFH: Unfractioned heparin; LMWH: Low-molecular-weight heparin; tPA: Tissue plasminogen activator; TFPI: Tissue factor pathway inhibitor; MRI: Magnetic resonance imaging.

## Competing interests

Non-financial competing interests.

## Authors’ contributions

All authors were involved in the management of the patient. All authors contributed to the draft and revision of the manuscript and approved the final manuscript.
